# Climate-Driven Ichthyoplankton Drift Model Predicts Growth of Top Predator Young

**DOI:** 10.1371/journal.pone.0079225

**Published:** 2013-11-12

**Authors:** Mari S. Myksvoll, Kjell E. Erikstad, Robert T. Barrett, Hanno Sandvik, Frode Vikebø

**Affiliations:** 1 Institute of Marine Research, Bergen, Norway; 2 Norwegian Institute for Nature Research, FRAM – High North Research Centre for Climate and the Environment, Tromsø, Norway; 3 Department of Natural Sciences, Tromsø University Museum, Tromsø, Norway; 4 Centre for Conservation Biology, Department of Biology, Norwegian University of Science and Technology, Trondheim, Norway; 5 Institute of Marine Research, Bergen, Norway; Dauphin Island Sea Lab, United States of America

## Abstract

Climate variability influences seabird population dynamics in several ways including access to prey near colonies during the critical chick-rearing period. This study addresses breeding success in a Barents Sea colony of common guillemots *Uria aalge* where trophic conditions vary according to changes in the northward transport of warm Atlantic Water. A drift model was used to simulate interannual variations in transport of cod *Gadus morhua* larvae along the Norwegian coast towards their nursery grounds in the Barents Sea. The results showed that the arrival of cod larvae from southern spawning grounds had a major effect on the size of common guillemot chicks at fledging. Furthermore, the fraction of larvae from the south was positively correlated to the inflow of Atlantic Water into the Barents Sea thus clearly demonstrating the mechanisms by which climate-driven bottom-up processes influence interannual variations in reproductive success in a marine top predator.

## Introduction

Climate change has a clear and significant impact across all ecosystems, latitudes and trophic levels with rates of change in phenology and distribution being faster among marine biota than on land [1]. Predicting the effects of climate variability on and through the different trophic levels is, however, a major challenge, and one that increases in complexity as one climbs the food chain. At the top of the marine chain are seabirds, the most numerous and visible of marine top predators. They have long been considered important sentinels of the marine ecosystem [2], [3] with effects of climate on life history traits being documented across many species and populations [4], [5]. Most seabird species are long-lived, produce small clutches and defer the onset of breeding. In terms of population change, the most influential life history traits are adult mortality rates, recruitment rates and offspring production. One of the main questions still raised is how climate influences population dynamics, or more specifically, what are the pathways along which climate variability filters to influence the different seabird life-history traits [6].

A seminal study in this context reported clear similarities between trends in long-term data series at four trophic levels in the North Sea (phytoplankton, zooplankton, herring *Clupea harengus* and black-legged kittiwakes *Rissa tridactyla*) and one of climate (westerly weather), but the mechanisms behind the similarity were unclear [7]. Later, Thompson and Ollason (2001) [8] showed how ocean climate variation had lagged effects on a Scottish pelagic seabird through cohort differences in recruitment related to temperature changes in summer. Frederiksen et al. (2006) [9] demonstrated consistent trends across four trophic levels in the North Sea (from plankton to seabirds), but again the causal links were undefined. Recent studies in contrasting climatic areas of the world have shown how climate change affects food availability and/or quality and hence breeding phenology of seabirds. In one study the change was advantageous to the species involved (royal penguin *Eudyptes schlegeli*, [10]) and in the other resulted in a trophic mismatch between five seabird species breeding in the North Sea and their prey (sandeels *Ammodytes marinus*, [11]) though, as yet, with no evidence of an impact on breeding success or population dynamics.

The common guillemot *Uria aalge* is a very common, circumpolar, boreal and low-Arctic seabird species [12]. The adults are long-lived and produce maximum one chick a year that is fed single individuals of small, energy-rich pelagic fish. The chicks have an intermediate fledging pattern and leave the breeding site when 15–21 d old and 15–35% of adult mass, long before they can fly. This is probably a result of several selection pressures resulting in a slow chick growth and low asymptotic mass [12]. Such pressures include the high flight costs of the adult with the energetic needs of the chick ultimately exceeding those which the adults can obtain and transport [13]. As such, the body condition of a fledging common guillemot chick not only reflects its own nutritional state but also that of its parents and the effort required by the parents to provision the chicks [14]. In the southern Barents Sea chicks are fed mainly capelin *Mallotus villosus*, herring *Clupea harengus* and sandeels *Ammodytes* sp. [15] while the adults feed largely on the youngest age-classes (20–80 mm in length) of Gadidae, such as northeast arctic (NEA) cod *Gadus morhua* or haddock *Melanogrammus aeglefinus* [16]. Whereas increases in 1-group herring abundance in the southern Barents Sea have been associated with higher chick fledging masses [17], this same and a later study have also emphasized the importance 0-group NEA cod in this area as adult food [18]. The abundance of 0-group cod prior to the breeding season is a major driver of the interannual variation in the breeding population growth while later in the season there is a probable close relationship between 0-group cod abundance and adult body condition (and hence their ability to feed chicks) [17], [18].

**Figure 1:**
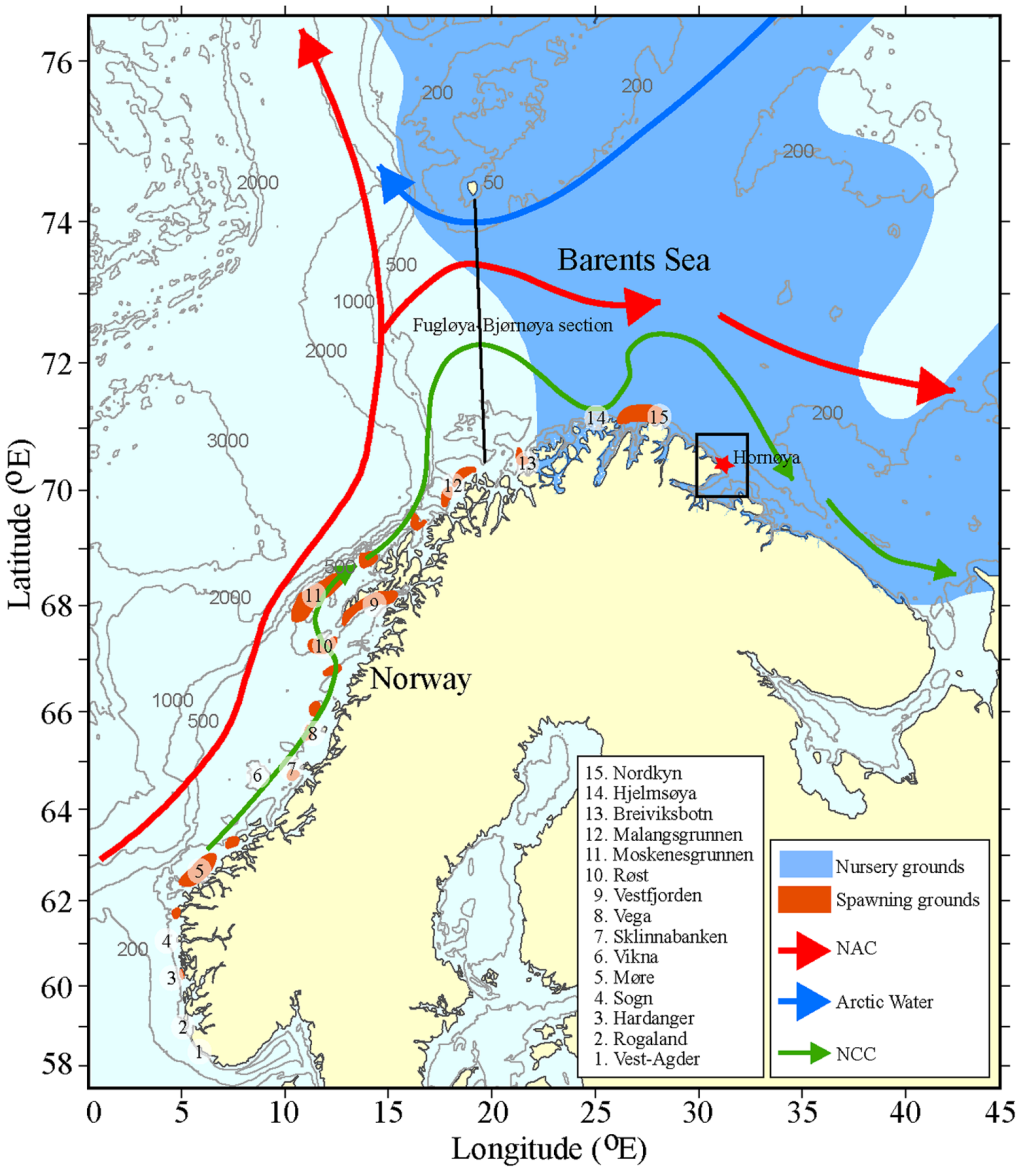
The study area. The study area including the most important current features, the Norwegian Atlantic Current (NAC) and the Norwegian Coastal Current (NCC), cod spawning areas numbered from south to north, the oceanographic section Fugløya-Bjørnøya and the common guillemot colony at Hornøya (red star) centered in the approximate foraging area of chick-feeding adults (black box).

The Barents Sea is a highly productive shelf ocean where the local variability in production is highly dependent on the transport of water from the Norwegian Sea [19]. This transport is either by the nearshore Norwegian Coastal Current (NCC) [20], which carries Coastal Water (CW), or by the offshore Norwegian Atlantic Current (NAC) [21], which carries Atlantic Water (AW) (Fig.1). North of Tromsøflaket, the NAC splits into two branches, one flowing east into the Barents Sea and the other continuing north towards Spitsbergen. The Barents Sea climate is thus the result of variations in temperature, salinity and volume of the inflow of the two water masses (AW and CW) [22], [23]. The climate conditions in turn affect the distribution and growth rate of ecologically important biological products such as zooplankton and fish larvae that are carried into the Barents Sea by these currents. For example, the early life stages of the NEA cod are transported into the Barents Sea from their spawning areas along the Norwegian coast, together with their main prey *Calanus finmarchicus* [24]. This transport has been described through numerous observations at discrete locations of circulation and hydrographic features of the ocean dynamics along the coast, and recently by an ocean drift model that enables us to describe and track individual cod eggs and larvae along the coast [25].

The objective of this study was to investigate fine-scale variations in temporal and spatial distribution of cod larvae as prey for common guillemots by using a larval drift model. Models can now document the climate effect on larval drift along the Norwegian coast and quantify the spatiotemporal availability. By recording the life history of NEA cod larvae arriving to seabird colonies we develop a unique scenario in which causal links between climate and chick growth can be directly traced. This study addresses the direct links between the physical effects of climate variability, the drift, accumulation and availability of food items along the Norwegian coast and the growth of common guillemot chicks in the southern Barents Sea.

## Methods

### Ethics Statement

Access to the study site was annually approved by the Finnmark County authority. Capture and measurements of the birds were licensed by the Norwegian Directorate for Nature Management (Ringing permit: RTB, 131) and were carried out according to Norwegian ethics regulations and European Council guidelines for Laboratory Animal Science.

### Individual-Based Model (IBM) for Cod Eggs and Larvae

Data from an ocean model archive established using the model ROMS (Regional Ocean Modeling System, www.myroms.org) covering the Nordic Seas were used as input to an individual-based model (IBM) of the drift and development of early life stages of cod (egg, larvae and juveniles) [26], [27]. The ocean model has a horizontal resolution of 4 km and contains daily averaged fields of temperature, salinity, currents and turbulence at 30 sigma-levels (terrain-following vertical coordinates) throughout the Nordic and Barents Seas. The total volume of water transported through the Fugløya-Bjørnøya section was calculated using the model archive, where AW transported with the NAC is defined by temperature >3°C and salinity >34.9 and CW transported with the NCC is defined by salinity <34.9 south of 72° 30′N [23]. AW is normally characterized by salinity >35 but due to a known bias in the model this criterion was reduced to 34.9 (Vidar Lien, pers. comm.). Two studies have shown that the ocean model is able to replicate observed currents and hydrography along the Norwegian Coast [26], [27]. The ocean data archive used here is based on an updated version of the model setup described in the papers listed above, but with higher resolution winds downscaled from the same data source (European Centre for Medium-Range Weather Forecasts, www.ecmwf.int).

In the model, particles representing cod eggs are released at 15 known spawning areas along the Norwegian coast (Fig. 1) and carried northwards with the ocean currents [28]. Northeast arctic cod spawn in March and April, with a peak in the beginning of April in Lofoten and up to two weeks later along the coast of Finnmark [29]. The IBM covers the whole spawning period and 4500 eggs were released every third day through March and April, in total 94500 eggs. The position, stage (egg or larvae), and temperature- and size-dependent growth of each particle are stored on a daily basis [30]. Here, the egg stage duration is fixed and set to three weeks [31]. Individual vertical positioning of eggs is modeled according to Thygesen and Ådlandsvik (2007) [32] based on measurements of egg buoyancy with mean 31.25 and standard deviation 0.69, in which salinity is equivalent to neutral buoyancy, and the modeled salinity and turbulence at the position of the egg. Larvae migrate vertically according to their swimming capability and light availability (see [31] for details). When running the model on an annual basis, an accumulation of particles within a 100×100 km box around Hornøya (Fig. 1) was regularly recorded during the breeding season of common guillemots. The actual distance from Hornøya to the edge of the box was between 46 and 72 km, within the foraging range (<100 km) of common guillemots [12] and similar to the 20–64 km range recorded using GPS-tracking on Hornøya in 2011 and 2012 (Signe Christensen-Dalsgaard, unpublished data).

### Field Data Common Guillemot

The study was carried out on Hornøya (70° 23′N, 31° 10′E), northeastern Norway, where ca. 10 000 pairs of common guillemots bred in 2010 (pers. obs.) and the analyses were based on the same chick and food data used in a previous study [17]. The guillemot chicks normally hatch in the second half of June and leave the ledges after about 15–21 days, thus while taking into account some interannual variability we define June and July as the guillemot chick-rearing season. Chicks were caught on their way from the nest site to the sea, weighed (±2.5 g), measured (wing length, from the carpal joint to the tip of the longest primary covert, ±0.5 mm) and ringed. Chick food data were collected by direct observation of food items given to chicks once a day on 4–12 days prior to the first night of chick weighing. Fuller details are given elsewhere [17], [33].

### Statistical Analysis

As a proxy of chick condition, we restricted our analyses to the wing length that increases almost linearly throughout the period the chick is on the breeding shelf [34]. As such this study investigates the effect of cod larval abundance off Hornøya on the somatic growth and approximate age of chicks as they leave the nest site. An earlier study has, however, shown that wing length and body mass of chicks at departure were also closely correlated (*R* ^2^ = 0.75, [17]) such that using the wing length in the analyses will also reflect body mass.

As a first step we tested for the presence of temporal linear trends in the body size of chicks, their diet and the environmental covariates that may influence the analyses. The fish variable diet is expressed as the fraction (arcsin transformed) and tested one by one for a temporal trend. Such a trend was found in the body size of chicks only (see Table S1) and these data were detrended before the analyses by estimating the residuals from a linear regression between each parameter and year. We then used GLMSELECT to determine the order of importance of explanatory variables. The GLMSELECT procedure is primarily a model selection procedure and does not include regression diagnostics or other post-selection facilities such as hypothesis testing or testing of contrasts. PROC GLMSELECT was used to select a model or a set of candidate models from a large number of variables and then further investigate details in other regression procedures. We then used an autoregressive model (AUTOREG) to test for heteroscedastic-consistent standard errors using the archtest option and to correct for any covariance in error structure over time. All analyses were carried out in SAS version 9.3 [35].

Multivariate linear regression models were used to examine the relationship between body size of chicks at fledging, the variability of inflow of Atlantic Water and the accumulation of cod larvae around Hornøya. As explanatory variables, we also included the fractions of fish species in the chick diet in different years (taken from [17]), the total spawning stock of capelin and the amount of 1-group of herring, common chick prey items for the guillemot in the present study colony [17]. Data on the spawning stock of capelin and 1-group herring were based on total estimates from the Barents Sea (acoustic and trawl surveys, [36]).

## Results

### Inflow of Atlantic Water

**Figure 2:**
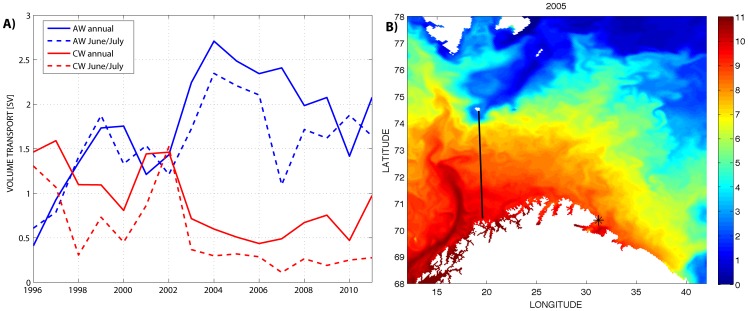
Volume transport and sea surface temperature in the Barents Sea. **A)** The volume of Atlantic Water (AW) and Coastal Water (CW) transported through the Fugløya-Bjørnøya section as calculated from the ocean model archive (1 Sv = 10^6^ m^3^s^−1^). **B)** The modeled sea surface temperature in the Barents Sea on 1 July 2005, the black line marks the Fugløya-Bjørnøya section.

**Figure 3:**
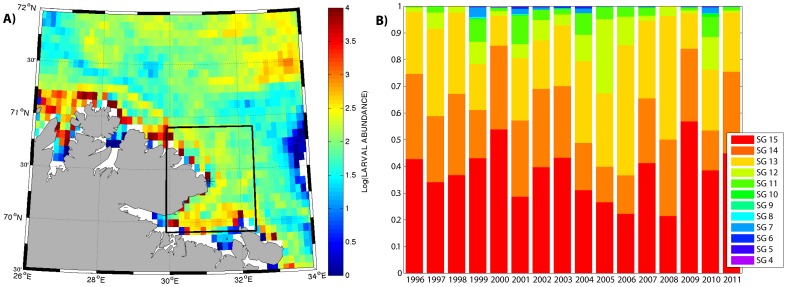
Original spawning grounds of cod larvae reaching Hornøya. **A)** Accumulation of cod larvae around Hornøya during July 2005. **B)** The original spawning grounds (SG) of cod larvae entering the foraging area of common guillemots (black box) around Hornøya during the approximate chick-rearing period 9 June to 7 July.

The mean annual and the mean inflow in June-July (the guillemot chick-rearing season) of Atlantic Water (AW) to the Barents Sea through the Fugløya-Bjørnøya section are shown in Figure 2A. The variability between years was large and the overall modeled AW transport during the period was 1.8 Sv (1 Sv = 10^6^ m^3^s^−1^). The summer inflow was generally weaker than the annual mean. The variable AW inflow through the Fugløya-Bjørnøya section has been shown to clearly reflect the climate variability in the Barents Sea [37], as illustrated in Figure 2B showing the sea surface temperature in the Barents Sea on 1 July 2005. The year 2005 was chosen as this was the year with highest fraction of cod larvae from southern spawning grounds reaching Hornøya (Fig. 3B). It is clear from the figure that the NAC transported heat far into the Barents Sea and towards Hornøya. The transport of CW through the section is also shown in Figure 2A, with an annual mean of 0.9 Sv, which was considerably weaker than the inflow of AW.

### Transport of Cod Larvae

Figure 3A shows the concentration of cod larvae accumulated through July in 2005 along the coast of northern Norway. The larvae were patchily distributed with highest concentrations near the coast and this pattern was similar between years. Cod larvae were sampled from the IBM within an estimated guillemot feeding range of Hornøya, represented by the black box in Figure 3A. Figure 3B shows which spawning grounds contributed to the larval concentrations near Hornøya every year from 1996 to 2011, specifically during the early chick-rearing period of common guillemots (9 June–7 July, unpubl. data). Spawning grounds 1 to 12 were named southern spawning grounds, as they were located south of the entrance to the Barents Sea (Fugløya-Bjørnøya section in Fig. 1). The origin of the larvae reaching Hornøya varied significantly between years and in some years the fraction of larvae from southern spawning areas was considerable, specifically 2005, 2010 and 1999. Most of the southern larvae reaching Hornøya were spawned at Malangsgrunnen and in the Lofoten region (Moskenesgrunnen, Røst and Vestfjorden), in addition to a small part from the Helgeland coast (Sklinnabanken and Vikna). Although fewer in numbers when they reach Hornøya, the larvae from the southern spawning grounds are on average 65% longer than those from the northern ones, 20.4 mm compared to 12.6 mm, and 23 days older. The southern spawned larvae thus represented a higher-quality food source for Hornøya guillemots as a consequence of their size that, in warm years, was additionally enhanced by their transport in warmer waters that provide better growth conditions. As such, each larva contributed proportionally more to the overall larval biomass than their northern counterparts. The fraction of cod larvae from southern spawning areas that accumulated around Hornøya during the chick-rearing period was positively correlated with the influx of AW through the Fugløya-Bjørnøya section in June and July (*R^2^* = 0.35, P = 0.02, Fig. S1). The influx of CW varied less and had no such effect (*R^2^* = 0.001).

### Effect on Chick Body Size

**Figure 4:**
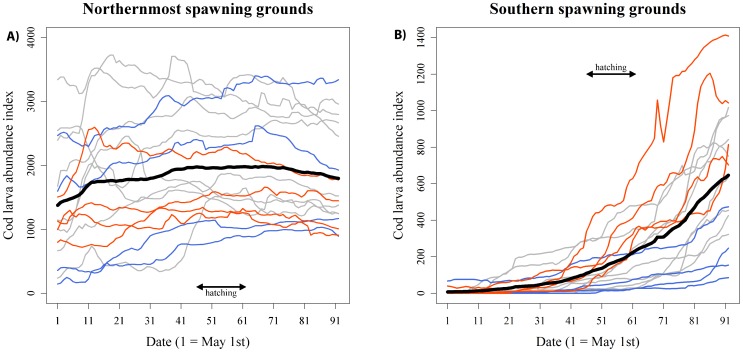
Arrival time of cod larvae to Hornøya. Accumulation of cod larvae from northern (**A**) and southern (**B**) spawning grounds around Hornøya during the breeding season of common guillemots in different years. Red lines mark the upper quartiles (4 years) of chick body size and blue lines the lower quartiles (4 years). Grey lines show the remaining eight years. The mean for all years is indicated by a bold line and the spread in guillemot hatching dates is indicated (unpubl. data). Note that the scaling on the y-axis differs in the two graphs.

**Figure 5:**
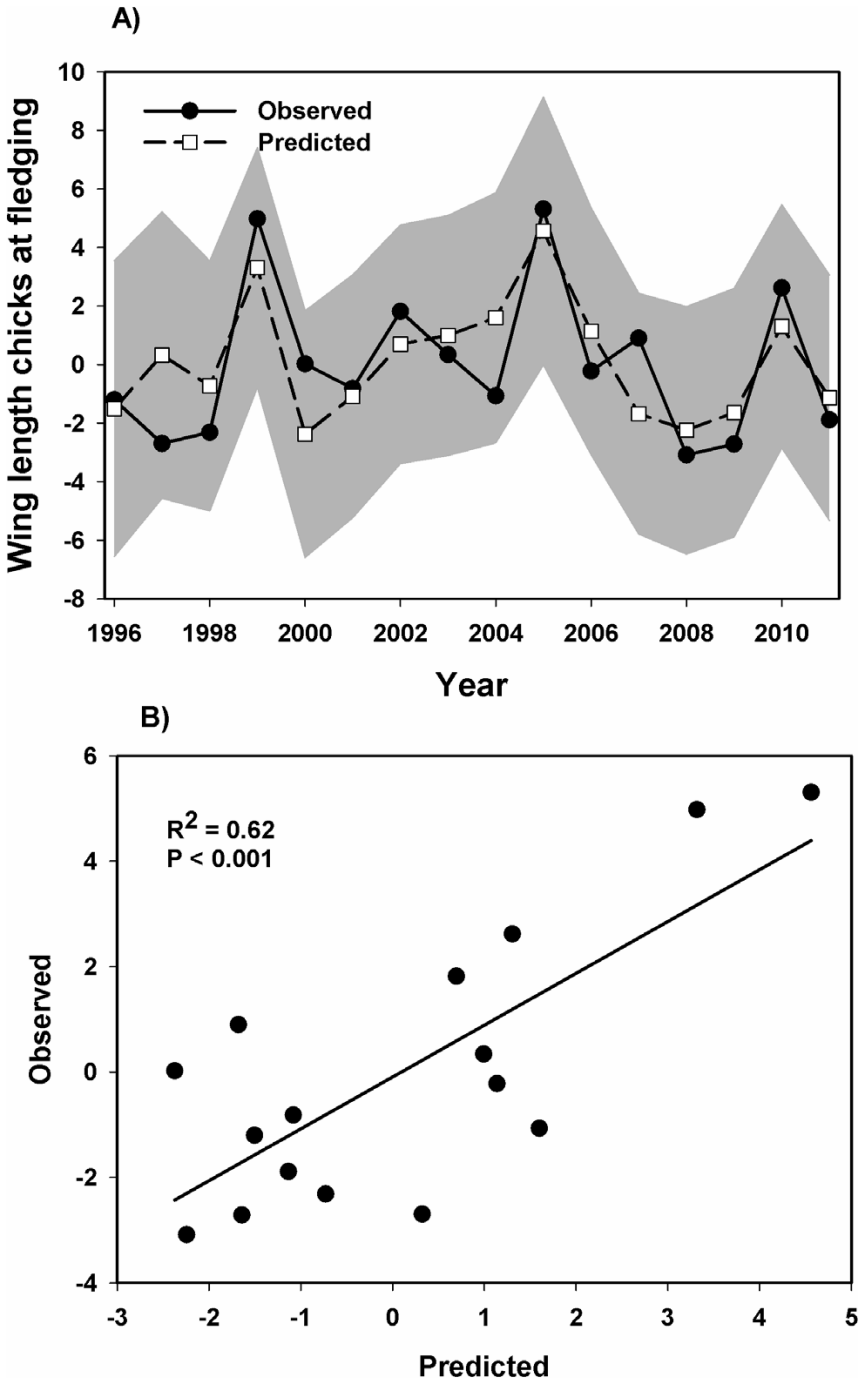
Annual variation in common guillemot chick body size. **A)** Annual variation in common guillemot chick body size at fledging and the fitted values from the autoregressive model between chick body size and the density of cod larvae from southern nursery grounds accumulating around Hornøya (with 95% confidence limits, shaded area) (Table 1, Tab. S1). **B)** The same data as in (A), with predicted values plotted against observed values.

Cod larvae from northern spawning grounds (Fig. 4A) were available to the guillemots for the whole breeding season, while larvae from southern spawning grounds (Fig. 4B) started to accumulate around Hornøya near the hatching date of the chicks. Using a set of explanatory variables in a backward procedure showed that the only variable that had any effect on the variation in chick body size at fledging was the fraction of cod larvae from the southern spawning grounds (Table 1, Tab. S1, Fig. 5). The concurrent arrival of southern larvae with the guillemots’ chick-rearing period explained the direct effect on the size of chicks leaving Hornøya early in the season with chicks being larger when larvae arrived early (Fig. 4B).

**Table 1:** Backstep selection model examining chick body size.

Step	Effect removed	Np	AICc	Adj R^2^	*P*
0		9	95.3	0.11	
1	Atlantic water	8	79.7	0.21	0.71
2	Population size	7	69.0	0.26	0.55
3	Capelin diet	6	61.4	0.29	0.48
4	Sandeel diet	5	55.1	0.34	0.62
5	Herring diet	4	50.7	0.36	0.45
6	Capelin	3	46.9	0.38	0.50
7	1-gr Herring	2	45.7	0.34	0.17
**Selected variables**		**df**	**Estimate±SE**	***t*** **- value**	**Pr >|** ***t*** **|**
Intercept		1	–1.92		
*Cod larvae*		1	0.00039±0.0001	2.94	0.01

A backstep selection summary (PROC GLMSELECT) from a model examining the relationship between the body size (wing length) of common guillemot chicks at fledging and a set of explanatory variables. The variables were 1) inflow of *Atlantic water* [SV], 2) *cod larvae* from southern spawning grounds reaching Hornøya, 3) ICES estimate of *1-gr herring* [logno × 10^6^] in the Barents sea, 4) ICES estimate of total spawning stock of *capelin* [logno × 10^6^] in the Barents Sea, 5) fraction of *capelin*, *herring* and *sandeel* [%] in the chick *diet* in different years and 6) the *population size* of common guillemots on Hornøya. Covariates were sequentially removed according to improvement of the model (the reduction of *AIC_C_* values). *Np*, number of parameters in the model, *Adj R^2^*, adjusted *R^2^* for the number of parameters in the model. Estimate and significance level are given for the selected parameter.

An autoregressive model (AR2 model) showed that the variance of density of cod larvae from southern spawning areas explained as much as 62% of the variation in chick body size over the years of this study (Table S2). Running the same model using chick body mass instead of body size (wind length) showed the same result, but with a lower explained variance (35%).

Although much of the adult diet includes young cod, the chicks were fed single fish of herring, capelin and sandeel, the contribution of which varied a lot between years (Fig. S2). The fraction of different fish species in the chick diets did not, however, explain any variation in chick body size (Table 1). There was a positive correlation between the fraction of 1-group herring in the diet and the influx of AW water (*R^2^* = 0.41, *P*<0.007) and a negative relationship between the fraction of adult capelin in the diet and the influx of AW (*R^2^* = 0.38, *P*<0.01) (Fig. S3).

## Discussion

### Main Findings

By combining an ocean model with an ichthyoplankton IBM for NEA cod we could show that variability in the relative contribution of cod larvae from southern spawning areas to waters near Hornøya was positively linked to the inflow of Atlantic Water (AW) to the Barents Sea. We also found that increased numbers of longer and hence higher-quality larvae were closely associated with common guillemot chicks being larger when they left the colony. Upstream southern water is relatively warm compared to the conditions farther north resulting in the larvae from southern spawning grounds being larger relative to their age than those from the northern grounds by the time they reach Hornøya.

Because the intermediate fledging pattern of guillemot chicks is thought to have evolved as a result of adults not being able to supply chicks with enough food to full fledging while in the colony [12], larger chicks can be interpreted as a sign of good feeding conditions near the colony for both the adults and the chicks. With plenty of food near the colony, the adults can afford to spend less time finding food for themselves, which on Hornøya consists mainly of 0-group NEA cod [16], and spend more time finding suitable fish for their chick. Before departure, the body mass increase of common guillemot chicks on Hornøya starts to taper off while the wing continues to grow [34]. As such, chicks leaving at an older age and thus longer wings will have an advantage over smaller chicks through better thermoregulatory abilities and a lower wing loading, resulting in an increase in the distance they can glide when jumping from the cliff.

Earlier studies have demonstrated the effect of AW inflow on the variability in the climatic conditions in the Barents Sea. We here show that it also explains much of the relative contribution of young cod from the different spawning grounds along the Norwegian coast to adult common guillemot prey near Hornøya and, consequently, the adults’ ability to provision chicks on the breeding ledge. At the same time, the inflow of AW is positively correlated with the occurrence of herring in the chick diet. Years with high AW inflow subsequently support both good conditions for the adults (cod larvae) and the chicks (herring). The prey availability for guillemots is therefore directly linked to variations in the physical environment. This study also demonstrates the vulnerability of the location of the colony at Hornøya due to its dependence on the non-local production of prey items and their variable transport routes. As they pass Hornøya, cod larvae are about to reach their final destination, the nursery grounds in the Barents Sea, and the residence time of each individual near the coast is strongly dependent on eddy activity [38]. While prevailing circulation features supply seabirds at Hornøya with cod larvae, eddy activity en route affects both the amount of supply and the local retention, i.e. temporal availability near the coast [38], [39]. Eddies are either semi-permanent, locked to topographic features or transient resulting from oceanic instabilities. Either way their occurrence can be expected to significantly contribute to within season and between year variability in the availability of cod larvae in the vicinity of Hornøya. This case study is thus a clear demonstration of how bottom-up processes influence the interannual variations in fledging success and population growth rate in a long-lived seabird population [18].

Erikstad et al. (2013) [18] showed that 0-group cod abundance had a lagged (6 years) and un-lagged positive impact on guillemot population growth rate. In the present study we can explain the lagged effect as the influence of good quality cod larvae on the size of fledging chicks through the fitness of their parents that, in turn, will affect the chicks’ survival rates and their ability to recruit to the population 6 years later. The un-lagged effect is related to the adult return rate (resighting rate) to the colony each year. As the adult survival rate at Hornøya is high and near constant [40], the resighting rate probably reflects the part of the population that defers breeding in any year such that the presence of young cod near Hornøya might be an important determinant of the adults’ decision to breed or not to breed.

### Model Evaluations

The ocean model archive provides a good representation of the Norwegian Atlantic Current (NAC) in the Fugløya-Bjørnøya section when compared to field observations. The modeled AW transport during the period was 1.8 Sv, which was slightly higher than the 1.5 Sv documented earlier [23]. Measurements using current-meter moorings in the section have shown that the transport of AW varied seasonally with higher inflow in winter than in summer [23], which was also evident in the model results. Evaluating the transport in the NCC is more difficult considering the wide range of estimates reported in the literature, ranging from 0.8 Sv to 1.8 Sv compared to 0.9 Sv in the model [20], [41]. The horizontal resolution of the ocean model is 4 km, which means that bank structures are only partially resolved and this will have an impact on eddy generation and air-sea interactions [38]. The model setup is not designed for detailed studies of transport with the NCC, mainly due to a nudging of the sea surface salinity in the numerical ocean model towards the boundary conditions, which underestimates the presence of the buoyancy-driven coastally trapped NCC fueled by freshwater runoff from land [20].

In the individual-based model (IBM) for cod larvae used here, growth was calculated as a function of temperature and size with unlimited food resources. Individual larvae may, however, experience variable prey densities and corresponding changes in growth and survival [42], which will affect both the spatio-temporal distribution and the body condition. Bugge et al. (2011) [16] reported that adult guillemots fed on 0-group cod within the size range 20–80 mm, which is somewhat longer than reported here. There are, however, limitations with the IBM, as mentioned above, especially since mortality is not included. As the individuals who grow fast are more likely to survive, the model will in general underestimate the size of larvae. Furthermore, the seabirds will most probably select the largest fish larvae such that the lengths reported by Bugge et al. (2011) [16] quite likely did not represent the total size range of larvae in the area.

### Ecological Considerations

While transporting cod larvae along the coast, the AW inflow to the Barents Sea is also a proxy for the flux of accompanying zooplankton, specifically *Calanus finmarchicus* [24]. Furthermore, Ottersen and Loeng (2000) [43] showed a link between temperature and year-class strength of cod, haddock and herring in the Barents Sea, with a direct effect on development rate and an indirect effect through prey availability. Hence, a strong inflow of AW might also lead to enhanced growth and survival of larvae and 0-group stages as they enter the Barents Sea, in addition to the increase in fraction of individuals from southern spawning grounds. While this issue cannot be addressed in the model system used here, it does suggest that the positive effects of increased AW inflow are larger than the model proposes.

The interannual variation in the composition of common guillemot chick diet at Hornøya is shown in Barrett and Erikstad (2013) [17]. Using the drift model in the present study we found a positive correlation between the influx of AW to the Barents Sea and the amount of 1-group herring in the chick diet. Note, however, that there was no relationship between the fraction of herring in the diet and chick mass (but see [17] for possible explanations). Herring may nevertheless feed on cod larvae [44], and with both herring (as chick food) and cod larvae (as adult food) available for the guillemots in the same area, it seems that warm years would be highly advantageous. Furthermore, we found a negative correlation between influx of AW and fraction of capelin in the chick diet. The distribution of capelin is known to shift to the northeast in warm years with high AW inflow making them less available around Hornøya [45].

We assume here that improved conditions for adult feeding and thus increased adult fitness result in chicks being fed more, staying longer in the colony before fledging and becoming more robust before they enter the sea. An argument against this is that it is an advantage to leave the colony as soon as possible into waters of low predator abundance [46] and thus avoid the dangers of predation by e.g. gulls *Larus* spp on the nest site. Such predation of chicks is, however, very rarely seen in dense colonies like the one from which our chicks departed (pers. obs., see also [47], [48], [49]).

The results of this study are fully consistent with earlier studies of the importance of the youngest stages of cod for the common guillemots that breed on Hornøya. Barrett and Erikstad (2013) [17] found a positive relationship between the amounts of 0-group cod in the waters around Hornøya and the fledging mass of chicks, while Erikstad et al. (2013) [18] demonstrated the importance of the youngest life stages of cod as a major driver of the breeding population. The present study is unique in that it reveals the direct bottom-up mechanism and the direct physical and climate drivers behind the variations in the quality of young cod in southern Barents Sea and the fledging condition of the chicks. Because the year class strength of NEA cod increases in warm years and the main spawning areas are known to shift up and down the Norwegian coast in response to water temperature [28], this model will be especially useful in predicting the effects of climate change on an important top predator in the Barents Sea.

## Supporting Information

Figure S1 **Relationship between inflow of Atlantic Water and cod larvae from south.** The relationship between annual variation in the influx of Atlantic Water to the Barents Sea and the proportion of cod larvae from southern spawning areas in the total cod larval concentration around Hornøya during the common guillemot breeding season.
(TIF)

Figure S2 **Composition of common guillemot chick diet.** Annual variation (by mass) in the composition of common guillemot chick diet at Hornøya, NE Norway (1996–2011). Annual sample sizes range from 390–1655 observations of adults with single-fish loads for chicks (Fig. 3 in Barrett & Erikstad 2013). The white solid line shows the variation in influx of Atlantic Water in different years.
(TIF)

Figure S3 **Relationship between inflow of Atlantic Water and herring/capelin in the chick diet.** The relationship between the fraction of herring (A) and capelin in the diet (B) of common guillemot chicks during the chick rearing period in relation to the variation in the influx of Atlantic water in different years.
(TIF)

Table S1 **Testing variables for linear trends.** Tests for annual linear trends in parameters used to examine the relationship between common guillemot chick body size (wing length) at Hornøya, NE Norway and various environmental factors. Sample size is 16 for all parameters (number of years from 1996 to 2011). Parameters with significant annual trends were normalized by using the residuals from a regression with year. The population size increased steeply over the years with an extraordinary good linear fit. There was therefore no need to detrend these data. The nearly perfect linear fit also precluded separating any trend in chick body mass over years from that of any density-dependent effect of population size.
(DOC)

Table S2 **Relationship between chick body size and cod larvae from south.** Output from an autoregressive model describing the relationship between body size (wing length) of common guillemot chicks and the modeled density of cod larvae from southern spawning areas accumulating around Hornøya during the chick-rearing period. The data could be best fitted to an AR2 model. Explained variance is 0.62.
(DOC)
