# Supplementary figures and images for: Climate-Driven Ichthyoplankton Drift Model Predicts Growth of Top Predator Young
Source: PLoS One. 2013 Nov 12;8(11):e79225. doi: 10.1371/journal.pone.0079225 (PMC3827142; doi:10.1371/journal.pone.0079225)

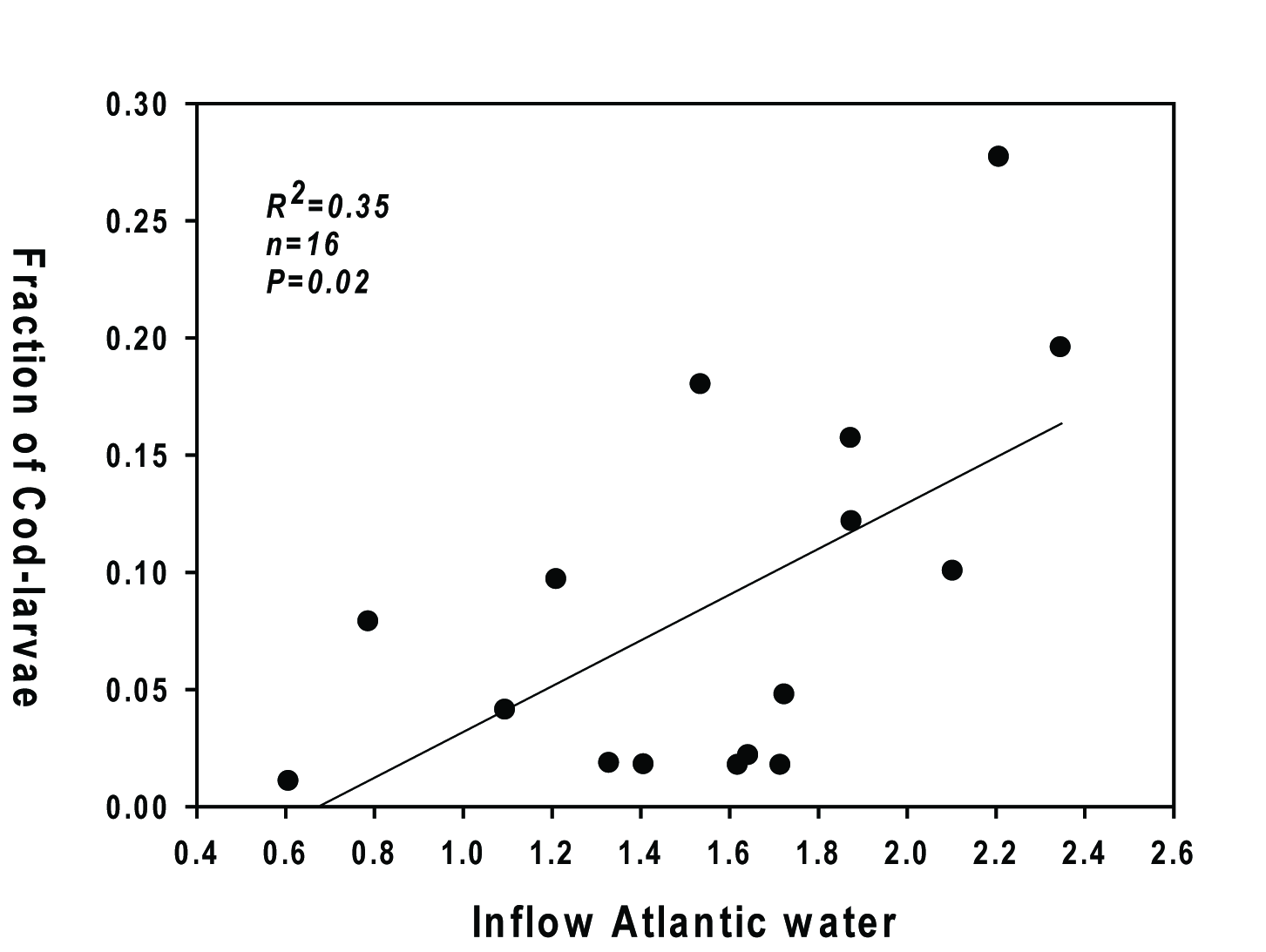

Supplement: Figure S1 — Relationship between inflow of Atlantic Water and cod larvae from south. The relationship between annual variation in the influx of Atlantic Water to the Barents Sea and the proportion of cod larvae from southern spawning areas in the total cod larval concentration around Hornøya during the common guillemot breeding season. (TIF) [file pone.0079225.s001.tif]

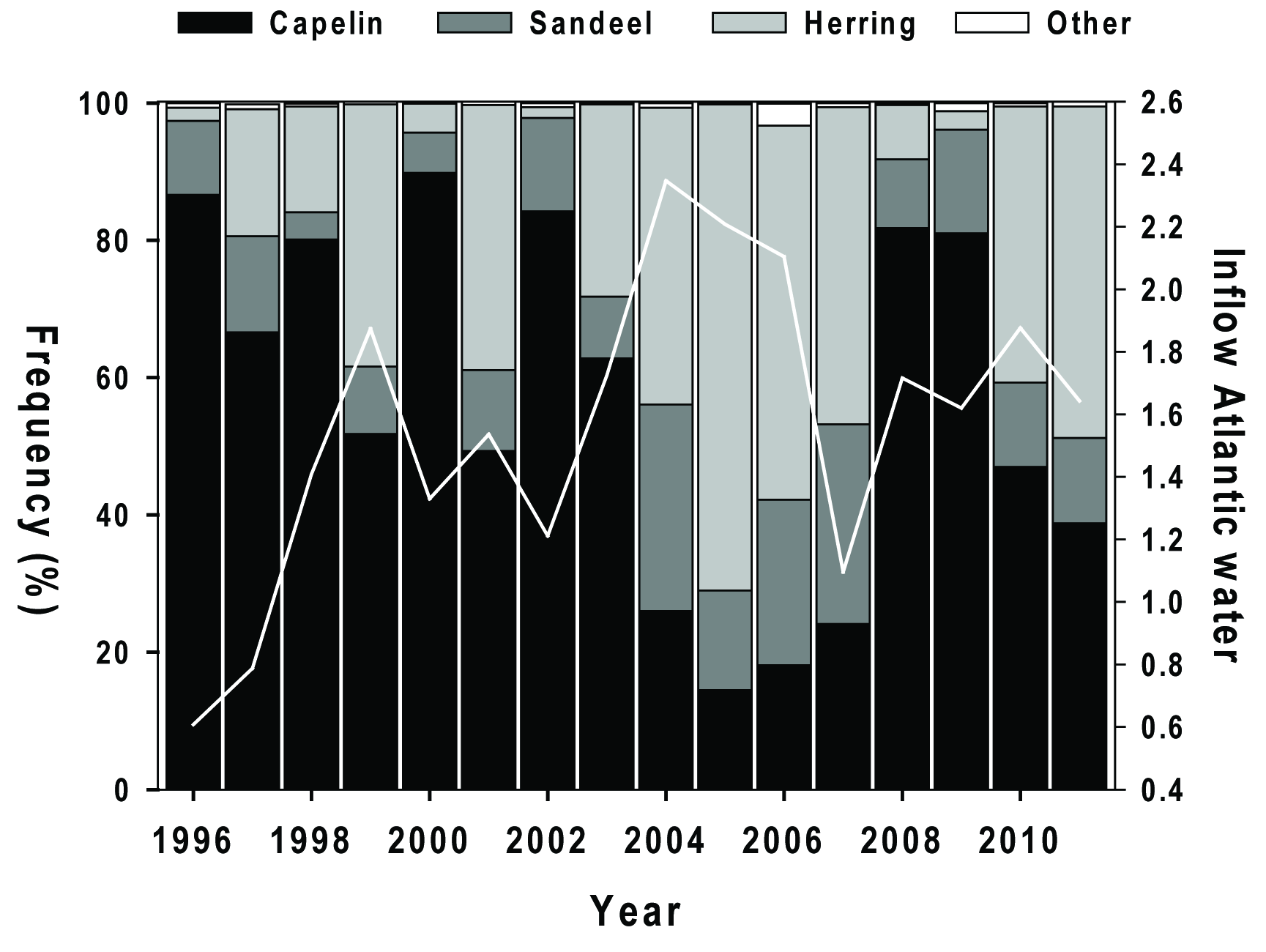

Supplement: Figure S2 — Composition of common guillemot chick diet. Annual variation (by mass) in the composition of common guillemot chick diet at Hornøya, NE Norway (1996–2011). Annual sample sizes range from 390–1655 observations of adults with single-fish loads for chicks (Fig. 3 in Barrett & Erikstad 2013). The white solid line shows the variation in influx of Atlantic Water in different years. (TIF) [file pone.0079225.s002.tif]

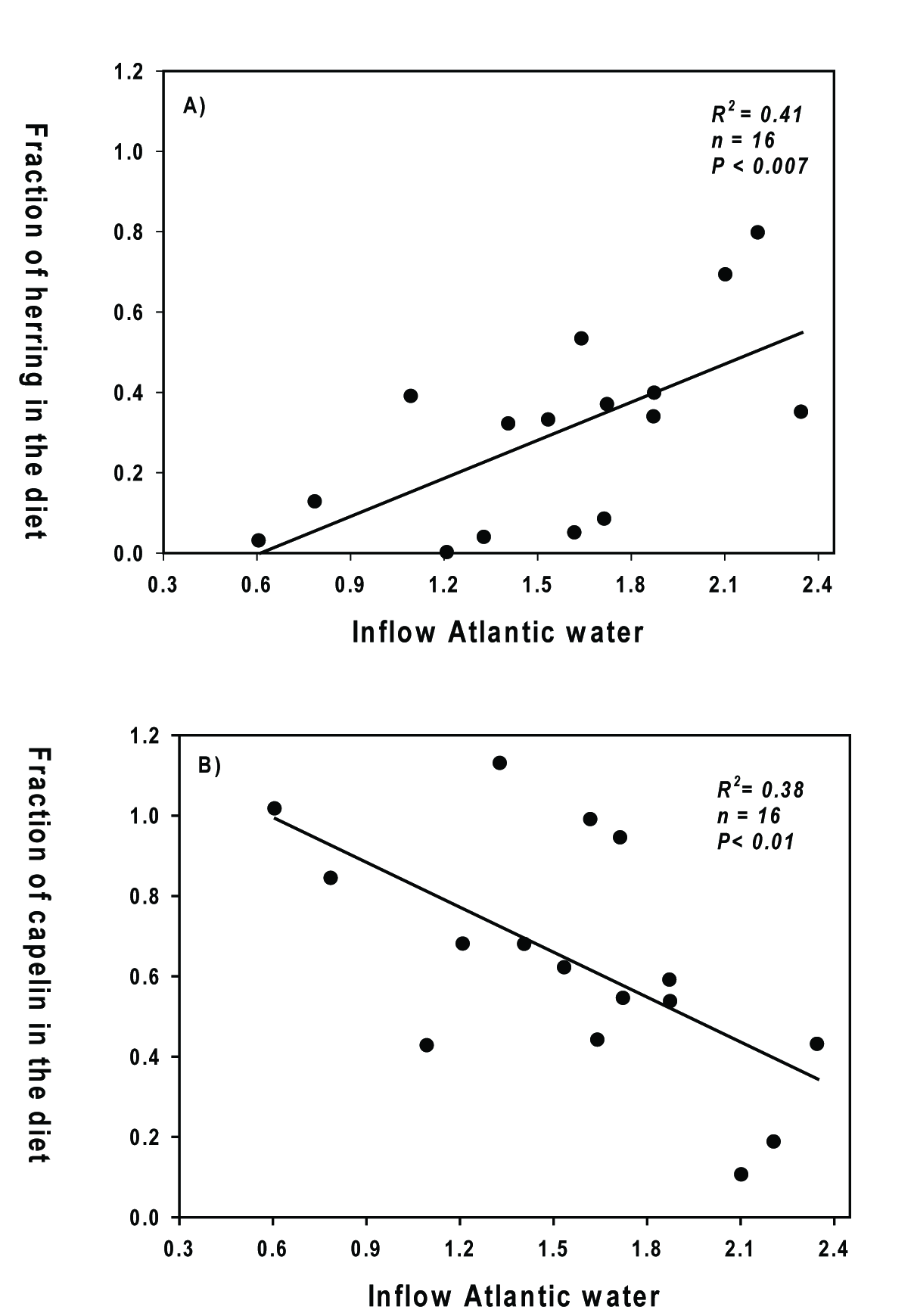

Supplement: Figure S3 — Relationship between inflow of Atlantic Water and herring/capelin in the chick diet. The relationship between the fraction of herring (A) and capelin in the diet (B) of common guillemot chicks during the chick rearing period in relation to the variation in the influx of Atlantic water in different years. (TIF) [file pone.0079225.s003.tif]
