# Supplementary material for: Climate-Driven Ichthyoplankton Drift Model Predicts Growth of Top Predator Young
Source: PLoS One. 2013 Nov 12;8(11):e79225. doi: 10.1371/journal.pone.0079225 (PMC3827142; doi:10.1371/journal.pone.0079225)
Supplement: Table S1 — Testing variables for linear trends. Tests for annual linear trends in parameters used to examine the relationship between common guillemot chick body size (wing length) at Hornøya, NE Norway and various environmental factors. Sample size is 16 for all parameters (number of years from 1996 to 2011). Parameters with significant annual trends were normalized by using the residuals from a regression with year. The population size increased steeply over the years with an extraordinary good linear fit. There was therefore no need to detrend these data. The nearly perfect linear fit also precluded separating any trend in chick body mass over years from that of any density-dependent effect of population size. (DOC) [file pone.0079225.s004.doc]

**Table S1.**

| Parameter | Slope (± SE) | *R^2^* | *P* | |  |
| --- | --- | --- | --- | --- | --- |
| Chick body mass | -1.83 (0.90) | 0.23 | 0.06 | | |
| Chick body size | -0.31 (0.15) | 0.25 | 0.05 | | |
| Cod larvae from south |  |  |  | |  |
| Atlantic water inflow (AW) | 0.05 (0.02) | 0.26 | 0.04 | |  |
| Coastal water inflow (CW) | -0.02 (0.008) | 0.26 | 0,04 | |  |
| Capelin | 0.12 (0.07) | 0.18 | 0.10 | |  |
| 1-group Herring | -0.05 (0.06) | 0.04 | 0.42 | |  |
| Herring in diet | 0.02 (0.01) | 0.14 | 0.16 | |  |
| Capelin in diet | -0.02 (0.02) | 0.13 | 0.17 | |  |
| Sandeel in diet | 0.0005 (0.005) | 0.12 | 0.91 | |  |
| Pop. size | 0.09 (0.003) | 0.99 | <0.001 | |  |
|  |  |  |  |  | |
