# Supplementary material for: Climate-Driven Ichthyoplankton Drift Model Predicts Growth of Top Predator Young
Source: PLoS One. 2013 Nov 12;8(11):e79225. doi: 10.1371/journal.pone.0079225 (PMC3827142; doi:10.1371/journal.pone.0079225)
Supplement: Table S2 — Relationship between chick body size and cod larvae from south. Output from an autoregressive model describing the relationship between body size (wing length) of common guillemot chicks and the modeled density of cod larvae from southern spawning areas accumulating around Hornøya during the chick-rearing period. The data could be best fitted to an AR2 model. Explained variance is 0.62. (DOC) [file pone.0079225.s005.doc]

**Table S2.**

| Variable | DF | Estimate +SE | *t-value* | *Pr >|t|* |
| --- | --- | --- | --- | --- |
|  |  |  |  |  |
| Intercept | 1 | -1.84 +0.51 |  |  |
| Cod larvae | 1 | 0.0004 + 0.00009 | 4.39 | 0.0009 |
| AR1 | 1 | 0.34 + 0.24 | 1.46 | 0.17 |
| AR2 | 1 | 0.58 + 0.24 | 2.41 | 0.03 |
